# Exploring Bioinformatics Solutions for Improved Leishmaniasis Diagnostic Tools: A Review

**DOI:** 10.3390/molecules29225259

**Published:** 2024-11-07

**Authors:** Natáli T. Capistrano Costa, Allana M. de Souza Pereira, Cibele C. Silva, Emanuelle de Oliveira Souza, Beatriz C. de Oliveira, Luiz Felipe G. R. Ferreira, Marcelo Z. Hernandes, Valéria R. A. Pereira

**Affiliations:** 1 Medicinal Theoretical Chemistry Laboratory, Department of Pharmaceutical Sciences, Health Sciences Center, Universidade Federal de Pernambuco, Recife 50670-901, PE, Brazil; natali.costa@ufpe.br (N.T.C.C.); emanuelle_oliveira01@outlook.com (E.d.O.S.); felzrt@gmail.com (L.F.G.R.F.); marcelo.hernandes@ufpe.br (M.Z.H.); 2 Immunopathology and Molecular Biology Laboratory, Departament of Immunology, Aggeu Magalhaes Institute, Recife 50740-465, PE, Brazil; cibele.carine@ufpe.br; 3 Lerner Research Institute, Clevealand, OH 44196, USA; coutinb@ccf.org

**Keywords:** leishmania, tropical disease, computational diagnosis, epitopes, immunoinformatics

## Abstract

Significant populations in tropical and sub-tropical locations all over the world are severely impacted by a group of neglected tropical diseases called leishmaniases. This disease is caused by roughly 20 species of the protozoan parasite from the *Leishmania* genus. Disease prevention strategies that include early detection, vector control, treatment of affected individuals, and vaccination are all essential. The diagnosis is critical for selecting methods of therapy, preventing transmission of the disease, and minimizing symptoms so that the affected individual can have a better quality of life. Nevertheless, the diagnostic methods do eventually have limitations, and there is no established gold standard. Some disadvantages include the existence of cross-reactions with other species, and limited sensitivity and specificity, which are mostly determined by the type of antigen used to perform the tests. A viable alternative for a more precise diagnosis is the application of recombinant antigens, which have been generated using bioinformatics approaches and have shown increased diagnostic accuracy. This approach proves valuable as it spans from epitope selection to predicting the interactions within the antibody–antigen complex through docking analysis. As a result, identifying potential new antigens using bioinformatics resources becomes an effective technique since it may result in an earlier and more accurate diagnosis. Consequently, the primary aim of this review is to conduct a comprehensive overview of the most significant in silico tools developed over time, with a focus on evaluating their efficacy and exploring their potential applications in optimizing the selection of highly specific molecules for a more effective diagnosis of leishmaniasis.

## 1. Introduction

The leishmaniases are a group of parasitic, non-contagious, diseases classified as neglected tropical diseases. These conditions exhibit a worldwide distribution, with a notable prevalence documented in Africa, Asia, and the Americas [1]. The World Health Organization (WHO) reports that over 1 billion individuals currently inhabit regions endemic to this disease, emphasizing its critical status as a substantial public health challenge.

Leishmaniasis exhibits three primary clinical forms [2]. Visceral leishmaniasis (VL), also known as Kala-azar, is the most fatal form of leishmaniasis and is characterized by systemic infections that can cause damage to the liver and spleen, among other organs. On an annual basis, approximately 30,000 cases are expected [1]. The most common form of leishmaniasis, cutaneous leishmaniasis (CL), is characterized by the development of skin lesions and affects approximately one million individuals each year [3]. Nevertheless, if not appropriately treated, it may develop into a third, more severe form known as mucocutaneous leishmaniasis (MCL), which can be identified by mucosal infiltration and nasal ulcers [4]. Multiple factors, including the host’s immune system, nutritional status, genetic background, environment, and the parasite species responsible for the infection, impact these clinical manifestations [5].

Approximately 20 species with the potential to infect humans have been described. Due to the broad spectrum of clinical symptoms and infective species, there is a scarcity of immunological data, making it challenging to fully comprehend the multiple types of immune responses [6]. Nevertheless, it has been documented that upon entry of the parasite into the circulatory system, innate immune cells are mobilized, triggering multiple regulatory, susceptibility, and/or resistance mechanisms. This, in turn, elicits a complex immune response encompassing both cell-mediated responses and the production of antibodies [7].

Disease prevention is facilitated through early diagnosis, vector control, treatment of affected individuals, and vaccination [8]. Early diagnosis is crucial to determine appropriate treatment strategies, halt disease progression, and alleviate symptoms, thereby improving patient quality of life [9]. While serological diagnosis techniques are prominent in leishmaniasis diagnosis, they are hindered by limitations such as cross-reactions with other species, low sensitivity, and specificity [10]. These limitations are largely influenced by the type of antigen used in the assays.

Techniques facilitating the identification and selection of immunogenic molecules have emerged as viable alternatives to the challenging task of selecting the most effective antigens for serological approaches [11]. In this context, reverse vaccinology, initially developed by Pizza et al., introduced the concept of utilizing computational methods to anticipate the selection of potential molecules for immunological investigations, including vaccinations and diagnostic testing. Recombinant antigens offer a promising alternative for achieving more precise disease diagnosis. Generated using bioinformatics technologies, these antigens have demonstrated improved diagnostic accuracy [12].

Numerous investigations into leishmaniasis diagnosis have extensively employed bioinformatics tools, specifically to explore recombinant proteins and synthetic peptides [13,14]. These in silico methodologies are founded upon predicting potential antigenic/immunogenic epitopes. This approach, recognized for its relative simplicity, offers cost savings by reducing expenses associated with culture maintenance and minimizes variations in sensitivity and specificity observed in conventional serological methodologies [14]. Consequently, the identification of potential new recombinant antigens through bioinformatics resources emerges as a reliable strategy, promising earlier and more accurate diagnosis. The principal aim of this work is to present a comprehensive review of the various in silico tools available up to the present. It critically assesses the performance and effectiveness of these computational methods and investigates their potential for refining highly specific diagnostic targets. By highlighting the importance of these methods, this review illustrates how their application can facilitate the development of diagnostic tools with increased specificity. Such advancements have the potential to significantly enhance prognostic outcomes for individuals suffering from leishmaniasis, as well as other infectious diseases.

## 2. The Complexity of Anti-*Leishmania* Immune Responses

When the vector—female Phlebotomus and *Lutzomyia* spp., in the Old World and New World, respectively—introduces the promastigote form of the parasite into the host’s bloodstream during their blood meal, the immune response begins [2]. The primary players of the innate immune response in pathogen protection, neutrophils, and macrophages are immediately recruited. These cells perform a dual purpose since they are involved in both parasite elimination and pathogenesis [15]. Macrophages are effective against *Leishmania* in phagocytic and antibacterial processes. These cells can either promptly eliminate the parasite or create an environment conducive to its replication [16]. However, the parasite possesses the capability to modulate the complement system through virulence factors, thereby facilitating its infiltration into other phagocytic cells [17].

Although the immunological mechanisms of the disease are highly complex and diverse, it is evident that T cells primarily mediate the protective response to the infection [18]. The activation of naïve T cells can simulate the immunological environment induced by antigens. A type 1 response (Th1), which focuses on clearing the infection, develops due to cytokines such as IFN-γ and TNF-α released by CD4+ T cells. Conversely, anti-inflammatory cytokines like IL-4, IL-13, and TGF-β are released during the type 2 response (Th2), promoting parasite proliferation. However, the TH1/TH2 paradigm in humans is not well established [19]. Given the complexity of the human immune system and the broad clinical spectrum of the disease, establishing an immunological pattern for this population is challenging [11]. CD8+ T cells seem to respond differently depending on the form of leishmaniasis [20]. These cells can modulate immunopathology and promote lesion development in cutaneous leishmaniasis caused by *L. braziliensis* [20]. Conversely, in visceral leishmaniasis caused by *L. donovani* and *L. infantum* species, CD8+ T cells have a protective role by forming effective granulomas, which are crucial for parasite eradication in both murine and human models [21].

While some studies indicate that B-cells may contribute to the exacerbation of the disease, other research suggests they are critical for the resolution of the infection. Furthermore, studies have demonstrated a correlation between parasite load, chronicity of infection, and the severity of the humoral response. According to research analyzing the humoral response produced in mice infected with three different species of *Leishmania*, which were related to the cutaneous form of the disease, high levels of antibodies were found to be associated with increased disease severity [22,23]. In contrast, antibodies appear to play a protective role in VL endemic areas, as demonstrated by the high prevalence of healthy seropositive individuals [24].

## 3. The Implications of Early Detection in Leishmaniasis Diagnosis

Early disease detection, chemotherapy, vector control, and a potential vaccine represent the most effective strategies for controlling leishmaniasis in its forms and clinical manifestations [8]. For a better prognosis, an early diagnosis must be performed in a simple, rapid, and effective way. Leishmaniasis is currently diagnosed through the combination of a number of criteria, including clinical characteristics presented by the patient, and epidemiological and laboratory data [10].

Different laboratory tests, including serological, parasitological, and molecular methods, have been developed to diagnose leishmaniasis [25]. Despite this multitude of tests, defining one as ideal for diagnosing this disease remains difficult. The vast clinical spectrum of cutaneous lesions, which are susceptible to easy misdiagnosis when clinically evaluated alongside other diseases with a similar presentation, is one of the main problems [9]. *Leishmania* species diversity has a direct effect on test accuracy. Asymptomatic cases and even co-infection with the human immunodeficiency virus (HIV) are additional risk factors [7].

In this scenario, serological assays are routinely employed to identify parasite antigens and/or anti-*Leishmania* antibodies in samples from affected individuals [26]. However, the efficiency of these approaches varies and is strongly related to the type of antigen utilized, as well as the species present during the infection. As a result, affected individuals exhibit a wide range of clinical symptoms [27].

Recent research has shown that different *Leishmania* species’ soluble *Leishmania* antigens (SLAs) create a wide variety of effects, indicating that the antigen’s properties affect the results. When the *Leishmania infantum* SLA was evaluated, the sensitivity and specificity ranged from 0 to 96.7% and 63 to 100%, respectively [28]. Lower efficacy was reported with antigens from *Leishmania major* or *Leishmania braziliensis*, with sensitivity ranging from 1 to 87.5% and specificity ranging from 21.3 to 100% [13]. Furthermore, these tests continue to be ineffective in identifying asymptomatic patients, as well as patients in the early stages of infection and with low antibody titers [28].

On the other hand, a potent new strategy for enhancing the efficacy of traditional diagnostic tests has been developed through the incorporation of molecular biology methodology and in silico technologies. The use of these innovative methods has made possible the investigation and selection of a new class of molecularly defined antigens, resulting in the discovery of new molecules of multiple types (recombinant proteins, chimeric proteins, and peptides) as potential candidates for serological diagnosis [13,29]. Several studies have attempted to improve conventional methods, such as ELISA, by incorporating these new molecules, with satisfactory sensitivity and specificity results (Table 1).

The prospect of using an antigen capable of monitoring antibody titers in VL is critical due to the long-term persistence of anti-*Leishmania* antibodies even after treatment [29]. Therefore, the potential for choosing more suitable antigens for the clinical form and the species involved is a potent strategy, as they are essential elements for determining the disease in patients and, as a result, its treatment.

## 4. Immunoinformatics In Silico Approaches for the Prediction of B-Cell Epitopes

B-cell epitopes are classified as either continuous (linear B-cell epitopes), consisting of an amino acid sequence from the primary protein structure, or discontinuous (conformational B-cell epitopes), which consists of atoms from surface protein’s constituent amino acids that are brought together by the folding of the polypeptide chain [43]. The prevalence of conformational epitopes accounts for approximately 90% of B-cell epitopes, indicating that linear B-cell epitopes constitute the minority. Despite this, there is a predominant emphasis on linear B-cell epitopes due to their simpler predictability, requiring only the amino acid sequence as input. In contrast, predicting discontinuous B-cell epitopes necessitates knowledge of the complete three-dimensional protein structure [44,45].

One of the challenges in the identification of innovative immuno-diagnostic targets is the detection of the antigenic region capable of activating the body’s defense system. In this sense, computational methods through propensity measurements and machine learning algorithms can assist in the projection of epitopes in antigenic regions [46]. Table 2 presents the most pertinent methods available for free online access.

Hopp and Wood [47,63] developed the first continuous epitope prediction method. The authors assigned to each amino acid, in a sequence, the hydrophilicity scale on the propound that hydrophilic regions are principally located at the protein surface, and are possibly antigenic. This approach is a component of the propensity scale methods, which identify the locations of linear B-cell epitopes by correlating the antigenic characteristics of proteins with the physicochemical properties of amino acids [64]. Although the original tool developed by Hopp and Wood [47,63] is no longer available in its original form, its functions can still be accessed through the ProtScale website (https://web.expasy.org/protscale), which offers a modern platform for applying the hydrophilicity scale and other physicochemical properties to analyze and identify regions likely to be antigenic [65].

Instead of relying on individual attributes for propensity measurements, the BcePred tool [48] utilizes combinations of physical and chemical parameters to predict linear B-cell epitopes. According to its developers, BcePred functions similarly to BEPITOPE [66], which is currently unavailable (as of August 2024). Both tools are designed to identify potential continuous B-cell epitopes and discern patterns within individual proteins or across entire translated genomes, and they also provide lists of potential peptides for synthesis and testing. BcePred’s prediction accuracy was evaluated using a database of 1029 experimentally verified epitopes and 1029 random peptides, with accuracy ranging from 52.92% to 57.53%, depending on the parameters used. The highest accuracy of 58.70% was achieved by combining four amino acid characteristics: hydrophilicity, flexibility, polarity, and exposed surface [48].

In addition to the propensity scale methods, a novel approach has emerged in the field of epitope prediction, although it may exhibit limited performance: the amino acid scale method. Due to its low performance, the use of machine learning (ML) has been introduced in these methods. The beginning server, developed based on recurrent artificial neural networks (ANNs), was the ABCpred server [49]. This server provides predictions for B-cell epitope(s) in an antigen sequence by using artificial neural networks. Users can select window lengths of 10, 12, 14, 16, and 20 (upper limit) as the predicted epitope length when the epitope length is less than 20 amino acids, and then the program will complete the “missing” amino acids using the original protein sequence. The dataset used for training and testing of the ABCpred server, consisting of 700 B-cell epitopes and 700 non-B-cell epitopes (negative data), achieved an accuracy of 65.93% using recurrent neural networks. Galvani et al. [67], using the ABCpred server, identified linear B-cell epitopes from the LiHyT, LiHyD, LiHyV, and LiHyP proteins to construct the chimeric protein ChimLeish. The resulting ChimLeish antigen exhibited 100% sensitivity and specificity for diagnosing cutaneous leishmaniasis, surpassing the performance of individual synthetic peptides and the SLA (*L. braziliensis* antigenic extract).

Other tools may use a combination of ML algorithms, such as LBtope [50], which was developed using Support Vector Machine (SVM) and IBk (Instance-Based Learner k-Nearest Neighbor (kNN) algorithm), for example, utilizing an extensive dataset of B-cell epitopes and non-epitopes, totalizing 12,063 epitopes and 20,589 non epitopes, both obtained from the IEDB database (www.iedb.org). It is worth highlighting the innovation of including experimentally validated structures of non-B-cell epitopes in the dataset used to develop a prediction tool, resulting in accuracies ranging from approximately 54% to 86% through the utilization of various features. The ABCpred and LBtope methods consist of ANNs trained on similar positive data, B-cell epitopes, but differ on negative data, the non-B-cell epitopes. The negative data for ABCpred consist of the use of random peptides, which may possibly contain non-validated B-cell epitopes, while the negative data used for LBtope consist of experimentally validated and thus confirmed non-B-cell epitopes from IEDB.

In a similar way to LBtope, the SVMtrip [46] also uses the SVM machine learning approach, which is contrasted by the circumstance that SVMtrip combines the methods of tri-peptide similarity and propensity scores to predict continuous B-cell epitopes in standalone software, and in an online server. The prediction performance indicates that SVMTriP achieves a precision of 55.2% when considering epitope size, with the optimal and default setting being 20 amino acids in length. Regarding the ROC curves, SVMTriP (AUC = 0.702) presented a larger true positive. Incorporating both tri-peptide subsequence similarity and propensity significantly boosts the predictive accuracy for linear B-cell epitopes.

BepiPred 2.0 [68] offers standalone software and an online server for continuous B-cell epitope prediction and is established through a Random Forest algorithm that has been trained with structures of epitopes identified from antigen–antibody (Ag-Ab) complexes. The dataset for the development of the tool involved 649 Ag-Ab complexes, considering all non-antibody protein chains having atoms within 4Å radius of their respective antibody’s Complementary Determining Region (CDR). After removing the complexes with similar antigen sequences (>70% identical), the overall count of structures was reduced to 160 Ag-Ab complexes, from which 5 randomly selected structures were selected among the final evaluation set, while the remaining 155 structures were distributed on five groups for cross-validation and algorithm training. When compared to other prediction tools, BepiPred 2.0 presented the highest AUC value (0.62), followed by BepiPred 1.0 (0.57) and LBtope (0.54) [68]. In a recent study, BepiPred-3.0 [51] was introduced, marking a significant methodological advancement through the integration of sequence representations from the Evolutionary Scale Modeling (ESM-2) protein language model [69]. This enhancement yielded substantial improvements in the accuracy of both linear and conformational epitope predictions. BepiPred-3.0 has demonstrated significant advancements over its predecessors in predicting B-cell epitopes. When re-evaluated against five antigens from the BepiPred-2.0 external test set, BepiPred-3.0 exhibited a marked improvement in performance, with AUC values rising from 0.57 and 0.60 in BepiPred-1.0 and 2.0, respectively, to 0.74 in BepiPred-3.0. This trend continued across a broader set of 15 antigens from the IEDB external test set, where BepiPred-3.0 achieved substantial AUC performance gains of 10% and 14% compared to BepiPred-2.0. It is important to note that the evaluated performance of BepiPred-2.0 might have been overfitted due to the overlap between its training data and the external test set. However, the incorporation of ESM-2 protein language model embeddings in BepiPred-3.0 allows for better generalization to novel datasets, underscoring the model’s enhanced predictive capability.

In a study by Moreira et al. [70], BepiPred was utilized to successfully identify and select B-cell epitopes. Peptides Pep45 and Pep48, both individually and in combination, exhibited outstanding diagnostic accuracy in detecting IgG in dogs infected with *L. infantum*, with sensitivities ranging from 86.4% to 100% and specificities between 90% and 100%. Dhom-Lemos et al. [40] effectively utilized BepiPred (Version 1.0) to identify several promising B-cell epitopes within the repetitive region of *L. infantum* kinesin. The identified epitopes demonstrated superior diagnostic performance, achieving sensitivity rates of 92.86% and specificity of 100% in human samples, and 88.54% sensitivity and 97.30% specificity in canine samples. BepiPred (Version 1.0) was utilized by Medeiros et al. [71] to identify a linear B-cell epitope within the TryP protein from *L. braziliensis*. The ELISA results using this peptide demonstrated superior diagnostic performance, achieving an accuracy of 94.29%, compared to 89.29% for recombinant TryP (rTryP), 65.00% for soluble *L. braziliensis* antigen (sLba), and 37.14% for the immunofluorescence assay (IFA).

**Figure 1:**
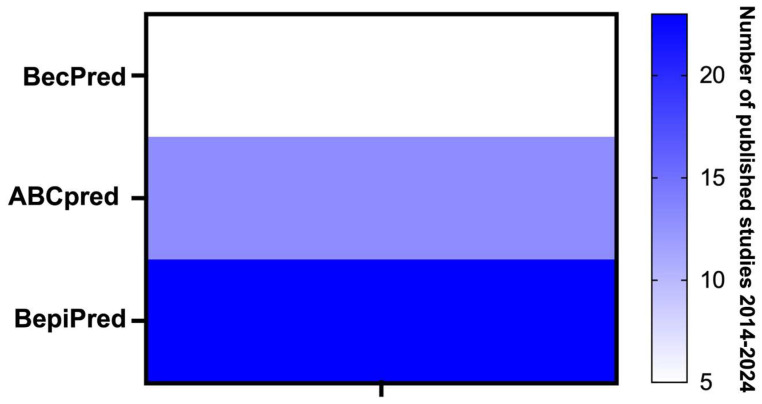
Most frequently employed in silico tools in the past decade for the selection of novel diagnostic molecules for leishmaniasis. Legend: This figure presents the distribution of published studies from 2014 to 2024, classified according to three B-cell epitope prediction tools: BecPred, ABCpred, and BepiPred. The color gradient on the right represents the number of studies, ranging from 5 to 20. The results demonstrate that BepiPred has the highest frequency of publications, followed by ABCpred, while BecPred has the lowest number of studies published within the given timeframe. Note: References for the works included and cited in the figure are provided in the Supplementary Materials.

Given the extensive array of tools available for preliminary studies—where the rationale and selection of the most promising targets for future diagnostic applications are determined (as detailed in Table 2)—it is apparent that this approach remains underutilized. Over the past decade, few of these tools have been employed to identify novel molecules that have advanced to the testing and evaluation phases for detecting individuals afflicted by the disease. Among these, BepiPred has emerged as the most prominent tool in recent research, as illustrated in Figure 1.

Predicting conformational B-cell epitopes has lagged behind linear epitope prediction, primarily due to two practical reasons. Firstly, it typically relies on having access to the three-dimensional structure of proteins, which is accessible to only a restricted quantity of proteins. Secondly, extracting conformational epitopes from their protein surroundings for targeted antibody production is a challenging task requiring appropriate scaffold structures. As a result, the prediction of conformational B-cell epitopes currently holds limited relevance in the context of designing epitope-based vaccines and antibody-based technologies. Nevertheless, it remains valuable for conducting structure–function studies related to antibody–antigen interactions [72]. The Conformational Epitope Prediction (CEP) server [52] uses a prediction method that, when tested using X-ray crystal structures of Ag-Ab complexes, accurately predicts discontinuous B-cell epitopes, antigenic determinants, and sequential epitopes with an accuracy of 75%. This tool is an advancement of the model “binding-determines function” that will aid in identifying the amino acids implicated in the Ag-Ab complex contact. Access to the CEP server is no longer available as of August 2024.

DiscoTope [53] is a tool capable of identifying approximately 15% of amino acids in discontinuous B-cell epitopes with a specificity exceeding 90%. DiscoTope innovates by combining information about amino acids, spatial location, and surface accessibility, which have been gathered on a dataset based on conformational epitopes established by X-ray crystallography of Ag-Ab complexes.

The ElliPro tool [54], named for its derivation from “Elli-psoid” and “Pro-trusion,” is a web-based application that employs three algorithms to approximate protein shapes in an ellipsoidal manner, based on their isoelectric point (pI) values, while clustering neighboring residues. This server incorporates a modified version of Thornton’s approach, which centers on the mass of each residue rather than its Cα atom [73]. The residue clustering is carried out using the MODELLER tool [74] and the resulting structures are visualized using the Jmol viewer (http://www.jmol.org) [75]. In a comparative analysis with other structure-based methods, ElliPro demonstrated the highest Area Under the Curve (AUC) value (0.732), followed by DiscoTope (0.601) and CEP (0.544), when considering the most significant prediction (best model generated) for each protein.

The SEPPA [55] server integrates the physicochemical properties of amino acids with their geometric structural characteristics. SEPPA (Spatial Epitope Prediction of Protein Antigens) introduced a new paradigm, "triangular residue unit patch", with the aim of improving epitope location prediction. SEPPA is now on version 3.0, facilitating glycoprotein antigens. When evaluated exclusively with independent glycoprotein antigens, SEPPA 3.0 demonstrated superior performance, achieving an AUC of 0.749 and a balanced accuracy (BA) of 0.665, outperforming its counterparts.

The EPITOPIA server [56] uses a machine learning algorithm that can handle both three-dimensional structures and sequence inputs to predict potential discontinuous B-cell epitopes. This approach uses a Naïve Bayes classifier on forty-four physicochemical and structural–geometrical attributes, including secondary structure, propensity, conservation, solvent-accessible surface, and hydrophilicity. When compared with ABCpred [49], which also has machine learning algorithms and received training on a very similar dataset, EPITOPIA [56] presented a better performance, yielding a success rate of 80.4% (AUC of 0.59), while ABCpred yielded a success rate of 67% (AUC of 0.55). When compared to other methodologies like DiscoTope [53] and ElliPro [54], EPITOPIA [56] presented a success rate of 89.4% against 81.8% and 80.3% for DiscoTope and ElliPro, respectively. Although CEP does not individually score amino acids, this server achieved a mean of 0.53 AUC, which was the lowest performance among the compared servers, with AUC results of EPITOPIA (0.6), DiscoTope (0.62), and ElliPro (0.59). The EPITOPIA server is no longer available as of August 2024.

In a study by Costa et al. [76], the ABCpred and EPITOPIA tools were used to identify conserved linear B-cell epitopes in hypothetical proteins. The synthesized peptides were then validated for their potential in diagnosing canine visceral leishmaniasis, with sensitivity values ranging from 81.93% to 97.59%, and specificity values between 78.14% and 85.12%.

In 2010, Ansari and Raghava [57] introduced CBTOPE (Conformational B-Cell Epitope Predictor), a pioneering tool for predicting conformational B-cell epitopes solely using the primary sequence of antigens, without relying on homology with known structures. CBTOPE innovatively introduced the concept of Composition Profile of Pattern (CPP), wherein the central residue interacts with the antibody within a sliding window. This tool is underpinned by Support Vector Machine (SVM) technology and is trained on physicochemical and sequence-derived features of conformational B-cell epitopes. CBTOPE boasts a prediction accuracy exceeding 85% and an impressive Area Under the Curve (AUC) value of 0.9.

In a study by Arab-Mazar et al. [77], antigenic B-cell epitopes were identified in accordance with the amino acid sequences of the GP63, LACK, and TSA proteins of *L. major,* utilizing the tools ABCpred, Bepipred, and LBTope. The findings indicated that *L. major’s* integrated recombinant GP63, LACK, and TSA multi-epitope antigens could be important components for constructing a viable diagnostic ELISA sandwich test for cutaneous leishmaniasis antigen detection. Menezes-Souza et al. [12] demonstrated that rLbMAPK3 and rLbMAPK4 might be among the target molecules for human and canine leishmaniasis immuno-diagnostics; linear B-cell epitopes were identified through the use of immunoinformatics tools, including the BepiPred program. Assis et al. [78] identified 148 linear B-cell epitopes using BepiPred and BcePred from the calpain-like cysteine peptidase (CP), thiol-dependent reductase 1 (TDR1), and HSP70 proteins of *L. infantum.* This study represented a pioneering effort by combining various computational epitope prediction methods, alongside an evaluation of secondary structures, to uncover epitopes associated with *Leishmania.*

The mimotopes approach represents a novel paradigm in B-cell epitope prediction, diverging from traditional methods by not solely relying on antigen information but also incorporating relevant data such as the sequences of the corresponding antibodies. Mimotopes, in this context, refer to peptides that mimic an epitope on the corresponding antigen, chosen from randomized peptide libraries for their ability to bind to a monoclonal antibody raised against a native antigen [79]. Several bioinformatics tools adopt the mimotope approach for predicting conformational epitopes. Examples available include EpiSearch [58] and MIMOP [59].

The mimotope approach presents a unique advantage by offering the ability to anticipate B-cell epitopes independently of the target protein sequence. This attribute allows for the identification of epitopes that may not remain consistent across various viral strains or species, proving exceptionally beneficial within the realm of infectious ailments. Additionally, the mimotope method shows potential in revealing novel immunogenic targets and expediting the development of pioneering vaccines and immunotherapies.

Deep learning techniques have recently emerged as powerful tools in the medical field, capable of identifying complex patterns within large datasets with greater accuracy and adaptability than traditional machine learning methods [80]. While various studies have explored the use of deep learning techniques in the study of protozoan parasites [81,82,83,84], these techniques have shown remarkable effectiveness in tasks like image classification, object detection, identification of insect vectors, and segmentation [85,86,87,88], making them highly suitable for diagnosing leishmaniasis from microscopic images. Deep learning enables precise identification of *Leishmania* amastigotes on stained slides, even in resource-constrained or remote environments [89,90,91,92,93,94,95]. Furthermore, innovative models, such as those based on GANs, have been developed to enhance image quality by correcting blurriness or focus issues, thereby improving diagnostic accuracy and advancing research in this area [96]. The use of these advanced technologies, including mobile applications, offers significant promise for improving leishmaniasis diagnosis, particularly in endemic regions with limited healthcare resources [96,97].

DeepLBCEPred is an advanced deep learning tool specifically developed for predicting B-cell epitopes from protein sequences. This method operates as an end-to-end system, meaning it starts with the raw protein sequence and directly produces a prediction. DeepLBCEPred has been rigorously tested on benchmark datasets, achieving top-tier performance, and is available as an easy-to-use web server, making it a valuable asset for researchers working in the field of immunoinformatics [60]. In a comparative analysis, DeepLBCEPred significantly outperformed LBtope and BepiPred, showing higher accuracy, increasing by 0.17 and 0.31, respectively, demonstrating superior specificity and sensitivity in predicting B-cell epitopes.

GraphBepi [61] is an innovative tool in B-cell epitope prediction, offering a fresh approach by combining structural and sequence information for greater accuracy. By utilizing AlphaFold2 [98] to predict protein structures and ESM-2 to capture sequence details, GraphBepi effectively integrates both spatial and contextual features. It employs an edge-enhanced deep graph neural network (EGNN) to interpret the structural data and a bidirectional long short-term memory network (BiLSTM) to grasp long-range dependencies within sequences. These elements work together to deliver precise predictions of B-cell epitopes. While the model’s reliance on AlphaFold2’s predictions is a current limitation, future improvements may include incorporating additional sequence-derived features and explainable models to enhance both its robustness and interpretability. GraphBepi significantly outperforms traditional methods in B-cell epitope prediction, achieving an AUC of 0.751, compared to 0.648 for Bepipred 2.0, 0.632 for ElliPro, and 0.635 for Discotope 2.0. This highlights GraphBepi’s superior accuracy in integrating both sequence and structural data.

SEMA 2.0 is a major advancement in predicting B-cell epitopes, built on the latest protein language models to deliver more precise results. This updated version employs ESM2 models, featuring 3 billion parameters, for enhanced sequence-based predictions and uses SaProt for accurate structure-based predictions, integrating both the antigen’s sequence and spatial arrangement. It also introduces new tools for predicting N-glycosylation sites and comparing antigen structures, which help identify similar epitopes and better understand their immunogenic properties. Compared to earlier tools like BepiPred 3.0 and SEPPA-3.0, SEMA 2.0’s advanced models and added functionalities offer a more comprehensive and effective approach to epitope prediction [62].

Despite endeavors to create novel epitope prediction algorithms, the field of bioinformatics research in this area still lacks software and servers capable of harnessing properties universally observed in antigenic epitopes, but not in other protein surfaces during predictive analyses.

## 5. Immunoinformatics in the Selection of Antibodies for Diagnosis

The Structural Antibody Database (SAbDab) is a web tool (available: https://opig.stats.ox.ac.uk/webapps/sabdab) database of antibody structures that has over 6000 antibody structures [99]. The SAbDab provides a variety of information about researched structures such as experimental information, details about genes and antigens, accurate heavy and light chain pairings, and in some cases, antibody–antigen binding affinity.

IMGT/mAb-DB is a unique database (available: https://imgt.org) dedicated to monoclonal antibodies within the IMGT^®^ framework, the globally recognized reference for immunogenetics and immunoinformatics. It serves as an invaluable resource for immunoglobulins (IGs), monoclonal antibodies (mAbs), and fusion proteins designed for immunological purposes, particularly those with therapeutic applications [100]. The server database contains 1261 entries, consisting of 1091 structures of immunoglobulin.

## 6. Immunoinformatics and Docking Analyses for Diagnostic Tools

Docking analyses reveal specific residue pairs at the interface, with the objective of enhancing binding affinity. This approach seeks to strategically enhance antigen complementarity on the antibody, thereby rationalizing improvements in the binding interaction [101]. Tools that use antibody-specific decoy generation and scoring methods perform better when compared with the general methods (protein–protein docking) [102]. Table 3 provides a summary of tools that incorporate specific algorithms for conducting antibody–antigen global docking and rigid body approaches.

The ClusPro server [108], a specialized tool for protein–protein docking, does not account for possible conformational changes upon binding (rigid body docking) and uses an algorithm derived from the Fast Fourier Transform (FFT) algorithm. FRODOCK [106] also employs FFT-based correlation algorithms but differs in its use of spherical harmonic (SH)-based rotational search, which has been demonstrated to be a faster process in protein–protein docking. PatchDock [104] employs a geometry-based molecular docking algorithm that integrates geometric hashing with pose clustering to elucidate interactions between antibody and antigen complexes. Its elevated efficiency could be explained because of the fast transformational search based on local feature matching, avoiding exhaustive orientation searches. ZDOCK [103] is a rigid protein docking program that performs a thorough search for probable binding modes of two-component proteins using FFT. This tool searches through each conceivable posture in the translation and rotation spaces of the two proteins. The ZDOCK uses an energy-based scoring function that ties together several parameters for scoring, such as calculating potential energy, spatial complementarity, and electric field force.

SnugDock [105] and HADDOCK [107] (Table 3) are tools that can perform flexible docking. The SnugDock is a Rosetta protocol (some of these protocols are fully automated via the ROSIE web server, https://rosie.rosettacommons.org) tailored to perform antibody–antigen docking. SnugDock’s local search algorithm models the CDR loops and the VH-VL orientation in the context of the antibody–antigen contact. When the crystallographic antibody’s structure is not accessible, this tool may predict high-resolution antibody–antigen complex structures, which is particularly helpful. The relative orientation of the antibody’s light and heavy chains, the conformations of the six complementarity determination region loops, and the placements of the antibody and antigen rigid bodies can all be optimized simultaneously using this method. Alternatively, the HADDOCK (High Ambiguity Driven protein–protein DOCKing) server [107] allows its users to perform protein–protein docking, taking into account the flexibility in the side chains and backbones, with the purpose of considering conformational rearrangements in the interaction surface. This tool combines a global rigid body search with ambiguous restraints, simulated annealing in torsion space, and minimization in Cartesian space.

Jeliazkov et al. [109] performed a comparative study of different docking tools, having specific options for antibody–antigen modeling, on sixteen target complexes. HADDOCK achieved a 75% success rate (based on the CAPRI quality criterion, possessing a model ranked within the top ten with acceptable or superior quality) [110] followed by ClusPro (67.8%) and ZDOCK (56.3%). In a recent evaluation involving 67 target complexes, Guest et al. [111] compared the effectiveness of ClusPro and ZDOCK. In an overall assessment, the findings indicated that both algorithms demonstrated similar performance on the benchmark. ClusPro exhibited slightly higher success rates, achieving a success rate of 34% on the benchmark, whereas ZDOCK generated a higher number of models with medium accuracy or better (22% success for ZDOCK compared to 16% for ClusPro).

Docking tools play a crucial role in immunoinformatics, particularly in the development and design of vaccines against *leishmania* [112,113,114]. In a recent study conducted by Onile et al. [115], an immunoinformatics analysis was carried out to propose a potential multi-epitope peptide vaccine for combatting fatal visceral leishmaniasis. The researchers employed three distinct online servers (ClusPro 2.0, PatchDock, and FireDock servers for refining PatchDock results) to evaluate the interaction between the multi-epitope peptide sequence and Toll-like receptor 4 (TLR4) (PDB ID: 4G8A) and Toll-like receptor 2 (TLR2) (PDB ID: 3A7C). Molecular docking analysis was utilized to assess the stability and affinity of this interaction, providing valuable insights into the potential efficacy of the peptide in eliciting an immune response. The docking outcomes were congruent with experimental assays, affirming the binding affinity and stability of the vaccine and TLR complexes.

In a separate investigation focused on devising a multi-epitope vaccine targeting *L. donovani* [116], the researchers employed the ClusPro Server to assess the interaction between the vaccine construct and Toll-like receptor 4 (TLR4) (PDB ID: 4G8A). The analysis unveiled the mode of interaction, highlighting the establishment of hydrogen bonds, salt bridges, and disulfide bonds between the vaccine construct and the TLR4 receptor. With a negative ΔG value of −13.3 kcal/mol and a dissociation constant (Kd) of 1.8 × 10^−10^, indicating energetically viable interactions with a high binding affinity, the study suggests the potential effectiveness of the vaccine in preventing or treating visceral leishmaniasis.

In a study conducted by Khatoon, Pandey, and Prajapati [117], the aim was to pinpoint immunogenic epitopes for crafting a multi-epitopic vaccine using immunoinformatics. The researchers selected the PatchDock server to execute protein vaccine-mediated targeted docking against TLR4, scrutinizing stability and binding affinities. The findings showcased the steadfastness of the formed complex, bolstering the prospect of employing the identified immunogenic epitopes in vaccine formulation.

Bioinformatics is a valuable resource for identifying new proteins and antigens that are suitable for use as targets for the diagnosis and treatment of infectious diseases. However, most studies on leishmaniasis focus on identifying new drugs and vaccines. While there are studies employing docking tools to discover potential *Leishmania* antigens, there is a shortage of studies documenting the utilization of these methods for the recognition of new diagnostic methods. Thus, studies in this area may be more challenging and may require greater experimental effort to validate docking results. Our research group has been applying the mentioned in silico tools, as evidenced by our publications in the field of immunoinformatics [118].

## 7. Conclusions

In conclusion, this review provides a comprehensive overview of current bioinformatics tools and trends aimed at enhancing their application. While the use of in silico strategies to identify recombinant antigens demonstrates significant potential to transform the diagnosis and effective control of leishmaniasis, thereby improving the quality of life for those infected, critical challenges remain. The efficacy of these computational tools is frequently dependent on the accuracy of the algorithms and the quality of the input data. Therefore, it is imperative that future research focuses on closing the gap between computational predictions and empirical validation. This approach is vital for ensuring that these tools can be effectively translated into reliable and practical diagnostic solutions. Continuous refinement and validation of these bioinformatics methods are essential for achieving their full potential in combating leishmaniasis and other infectious diseases.

## Figures and Tables

**Table 1:** Proteins and peptides selected in silico and their performance in ELISA for leishmaniasis diagnosis.

Peptide/Protein	Performance	Clinical Form	Reference
rCatL	SPE: 95.71% SENS: 95.56%	Visceral leishmaniasis	https://doi.org/10.1371/journal.pntd.0003426 [30]
rLiHyS	SPE: 100% SENS: 100%	Visceral leishmaniasis and cutaneous leishmaniasis	https://doi.org/10.1016/j.parint.2018.02.001 [31]
rPHB + synthetic peptide	rPHB SPE: 98.31% SENS: 84.91% synthetic peptide SPE: 98.31% SENS: 100%	Visceral leishmaniasis and cutaneous leishmaniasis	https://doi.org/10.1016/j.jim.2019.112641 [32]
rLbMAPK3 rLbMAPK4	rLbMAPK3 (LC) SPE: 71.43% SENS: 83.08% rLbMAPK3 (LV) SPE: 31.43% SENS: 94.55% rLbMAPK4 (LC) SPE: 97.14% SENS: 75.38% rLbMAPK4 (LV) SPE:88.57% SENS: 72.73%	Visceral leishmaniasis and cutaneous leishmaniasis	https://doi.org/10.1007/s00253-014-6168-7 [12]
rLbHyM	SPE: 98.0% SENS:100%	Cutaneous leishmaniasis	https://doi.org/10.1007/s00436-017-5397-y [33]
LbK39	(LC) SPE: 98% SENS: 88% (LV) SPE: 100% SENS: 100%	Visceral leishmaniasis and cutaneous leishmaniasis	https://doi.org/10.1016/j.onehlt.2019.100111 [34]
rLiHypA	SPE: 100% SENS: 98.2%	Cutaneous leishmaniasis	https://doi.org/10.1016/j.cellimm.2017.06.001 [35]
rLB8E e rLb6H	rLb6H SPE: 100.0% SENS: 98.5% rLb8E SPE: 83.3% SENS: 83.3%	Cutaneous leishmaniasis	https://doi.org/10.1128/jcm.01904-16 [28]
rLiHyC + synthetic peptide	rLiHyc + synthetic peptide: SPE: 100% SENS: 100%	Visceral leishmaniasis	https://doi.org/10.1016/j.actatropica.2019.105318 [36]
ChimLeish	SPE: 100% SENS: 100%	Visceral leishmaniasis	https://doi.org/10.1007/s00436-021-07342-1 [37]
rSMP-3	SPE: >90% SENS: >90%	Cutaneous leishmaniasis	https://doi.org/10.1016/j.imbio.2018.09.003 [38]
rEF1b	LC/LV: SPE: 100% SENS: 100%	Visceral leishmaniasis and cutaneous leishmaniasis	https://doi.org/10.1016/j.micpath.2019.103783 [39]
Synthetic peptides (P1, P2, and P3) and MIX(P1P2P3)	MIX SENS: 79% P1 SENS: 72% SPE: 78–100% P2 e P3 *	Visceral leishmaniasis	https://doi.org/10.1155/2017/5871043 [29]
rKDDR	SPE: 100% SENS: 92.86%	Visceral leishmaniasis	https://doi.org/10.1371/journal.pone.0211719 [40]
rK28	ESP: 98.6% SENS: >96%	Visceral leishmaniasis	https://doi.org/10.1186/s13071-016-1667-2 [41]
rLiHyV	SPE: 95.4% SENS: 85%	Cutaneous leishmaniasis	https://doi.org/10.1186/s13071-015-0964-5 [42]

Legend: SPE: specificity; SENS: sensibility; LC: cutaneous leishmaniasis; and LV: visceral leishmaniasis. * Unpublished data.

**Table 2:** Predictive methods for B-cell epitopes accessible online at no cost.

Linear B-Cell Epitope			
Tool	Method	Access	Reference
Hopp and Wood (1981) [47]	PS	https://web.expasy.org/protscale/, accessed on 20 August 2024.	https://doi.org/10.1073/pnas.78.6.3824
BcePred (2004) [48]	Combinations of physical and chemical parameters	http://crdd.osdd.net/raghava/bcepred/, accessed on 20 August 2024	https://doi.org/10.1007/978-3-540-30220-9_16
ABCpred server (2006) [49]	ML	http://crdd.osdd.net/raghava/abcpred/, accessed on 20 August 2024	https://doi.org/10.1002/prot.21078
SVMtrip (2012) [46]	ML	http://sysbio.unl.edu/SVMTriP/, accessed on 22 August 2024	https://doi.org/10.1371/journal.pone.0045152
LBtope (2013) [50]	ML	http://crdd.osdd.net/raghava/lbtope/ accessed on 20 August 2024	https://doi.org/10.1371/journal.pone.0062216
BepiPred 3.0 (2022) [51]	ML	https://services.healthtech.dtu.dk/services/BepiPred-3.0, accessed on 20 August 2024	https://doi.org/10.1002/pro.4497
**Conformational B-cell epitope**			
**Tool**	**Method**	**Access**	**Reference**
CEP (2005) [52]	Uses X-ray crystal structures of Ag-Ab complexes to predict conformational epitopes	http://bioinfo.ernet.in/cep.htm (*Unavailable as of August 2024*)	https://doi.org/10.1093/nar/gki460
DiscoTope (2006) [53]	Combines the propensity scale matrices, spatial proximity, and surface exposure	http://tools.iedb.org/discotope/, accessed on 22 August 2024	https://doi.org/10.1110/ps.062405906
ElliPro (2008) [54]	A modified version of Thornton’s approach	http://tools.iedb.org/ellipro/, accessed on 20 August 2024	https://doi.org/10.1186/1471-2105-9-514
SEPPA 3.0 (2009) [55]	Combines single physicochemical properties of amino acids with geometrical structural properties	http://www.badd-cao.net/seppa3/index.html, accessed on 20 August 2024	https://doi.org/10.1093/nar/gkp417
EPITOPIA server (2009) [56]	ML	https://epitopia.tau.ac.il (*Unavailable as of August 2024*)	https://doi.org/10.1186/1471-2105-10-287
CBTOPE (2010) [57]	ML	http://www.imtech.res.in/raghava/cbtope/, accessed on 20 August 2024	https://doi.org/10.1186/1745-7580-6-6
EpiSearch (2009) [58]	Mimotope	https://curie.utmb.edu/episearch.html, accessed on 20 August 2024	https://doi.org/10.4137/bbi.s2745
MIMOP (2006) [59]	Mimotope	Upon request	https://doi.org/10.1093/bioinformatics/btl012
**Deep learning techniques for prediction of B-cell epitope**			
**Tool**	**Type of B-cell Epitope**	**Access**	**Reference**
DeepLBCEPred (2023) [60]	Linear	http://www.biolscience.cn/DeepLBCEPred/, accessed on 20 August 2024	https://doi.org/10.3389/fmicb.2023.1117027
GraphBepi (2023) [61]	Linear and conformational	https://bio-web1.nscc-gz.cn/app/graphbepi, accessed on 20 August 2024	https://doi.org/10.1093/bioinformatics/btad187
SEMA 2.0 (2024) [62]	Conformational	https://sema.airi.net, accessed on 20 August 2024	https://doi.org/10.1093/nar/gkae386

Legend: PS: propensity scale; ML: machine learning.

**Table 3:** Docking tools for protein–protein interaction prediction.

Docking Tools			
Tool	Method	Access	Reference
ZDOCK [103]	Rigid protein docking program using FFT techniques	https://zdock.umassmed.edu, accessed on 20 August 2024	https://doi.org/10.1002/prot.10389
PatchDock (2005) [104]	Geometry-based molecular docking algorithm	http://bioinfo3d.cs.tau.ac.il/PatchDock/, accessed on 20 August 2024	https://doi.org/10.1093/nar/gki481
SnugDock (2010) [105]	Docking and the relative orientation of the antibody and antigen bodies can be optimized	https://rosie.rosettacommons.org/snug_dock, accessed on 20 August 2024	https://doi.org/10.1371/journal.pcbi.1000644
FRODOCK 2.0 (2016) [106]	Uses FFT correlation algorithms, with differences in spherical harmonic-based rotational search	https://frodock.iqf.csic.es, accessed on 20 August 2024	https://doi.org/10.1093/bioinformatics/btw141
HADDOCK (2016) [107]	Combines a global rigid body search with ambiguous restraints	https://wenmr.science.uu.nl/haddock2.4, accessed on 22 August 2024	https://doi.org/10.1016/j.jmb.2015.09.014
ClusPro 2.0 (2017) [108]	Algorithm based on the FFT	https://cluspro.bu.edu, accessed on 20 August 2024	https://doi.org/10.1038/nprot.2016.169

Legend: FFT: Fast Fourier Transform.

## Data Availability

All data supporting the findings of this review are available in the main article and its Supplementary Materials. For further information, please refer to the cited references.

## Supplementary Materials

The following supporting information can be downloaded at: https://www.mdpi.com/article/10.3390/molecules29225259/s1, References [12,30,33,36,40,70,71,78,119,120,121,122,123,124,125,126,127,128,129,130,131,132,133,134,135,136,137,138,139,140,141,142,143,144] are cited in the Supplementary Materials.

## References

[1] WHO/PAHO (2019). Leishmaniases: Epidemiological Report of the Americas. Department of Neglected Infectious Diseases. https://www.paho.org/en/topics/leishmaniasis.

[2] Sasidharan S., Saudagar P. (2021). Leishmaniasis: Where Are We and Where Are We Heading?. Parasitol. Res..

[3] Mokni M. (2019). Leishmanioses Cutanées. Ann. Dermatol. Venereol..

[4] Handler M.Z., Patel P.A., Kapila R., Al-Qubati Y., Schwartz R.A. (2015). Cutaneous and Mucocutaneous Leishmaniasis. J. Am. Acad. Dermatol..

[5] Hurrell B.P., Regli I.B., Tacchini-Cottier F. (2016). Different Leishmania Species Drive Distinct Neutrophil Functions. Trends Parasitol..

[6] Singh S. (2006). New Developments in Diagnosis of Leishmaniasis. Indian. J. Med. Res..

[7] Figueiredo M.M., dos Santos A.R.R., Godoi L.C., de Castro N.S., de Andrade B.C., Sergio S.A.R., Jerônimo S.M.B., de Oliveira E.J., Valencia-Portillo R.T., Bezerra L.M. (2021). Improved Performance of ELISA and Immunochromatographic Tests Using a New Chimeric A2-Based Protein for Human Visceral Leishmaniasis Diagnosis. J. Immunol. Res..

[8] Von Zuben A.P.B., Donalísio M.R. (2016). Dificuldades na Execução das Diretrizes do Programa de Vigilância e Controle da Leishmaniose Visceral em Grandes Municípios Brasileiros. Cad. Saude Publica.

[9] Aronson N.E., Joya C.A. (2019). Cutaneous Leishmaniasis. Infect. Dis. Clin. North Am..

[10] De Brito R.C.F., Aguiar-Soares R.D.d.O., Cardoso J.M.d.O., Coura-Vital W., Roatt B.M., Reis A.B. (2020). Recent Advances and New Strategies in Leishmaniasis Diagnosis. Appl. Microbiol. Biotechnol..

[11] Pizza M., Scarlato V., Masignani V., Giuliani M.M., Aricò B., Comanducci M., Jennings G.T., Baldi L., Bartolini E., Capecchi B. (2000). Identification of Vaccine Candidates Against Serogroup B Meningococcus by Whole-Genome Sequencing. Science.

[12] Menezes-Souza D., Mendes T.A.d.O., Leão A.C.d.A., Gomes M.d.S., Fujiwara R.T., Bartholomeu D.C. (2015). Linear B-Cell Epitope Mapping of MAPK3 and MAPK4 from Leishmania Braziliensis: Implications for the Serodiagnosis of Human and Canine Leishmaniasis. Appl. Microbiol. Biotechnol..

[13] Souza A.P., Soto M., Costa J.M.L., Boaventura V.S., de Oliveira C.I., Cristal J.R., Barral-Netto M., Barral A. (2013). Towards a More Precise Serological Diagnosis of Human Tegumentary Leishmaniasis Using Leishmania Recombinant Proteins. PLoS ONE.

[14] Duarte M.C., Pimenta D.C., Menezes-Souza D., Magalhães R.D.M., Diniz J.L.C.P., Costa L.E., Chávez-Fumagalli M.A., Lage P.S., Bartholomeu D.C., Alves M.J.M. (2015). Proteins Selected in Leishmania (Viannia) Braziliensis by an Immunoproteomic Approach with Potential Serodiagnosis Applications for Tegumentary Leishmaniasis. Clin. Vaccine Immunol..

[15] Oualha R., Barhoumi M., Marzouki S., Harigua-Souiai E., Ben Ahmed M., Guizani I. (2019). Infection of Human Neutrophils With Leishmania Infantum or Leishmania Major Strains Triggers Activation and Differential Cytokines Release. Front. Cell Infect. Microbiol..

[16] Serrano-Coll H., Cardona-Castro N., Ramos A.P., Llanos-Cuentas A. (2021). Innate Immune Response: Ally or Enemy in Cutaneous Leishmaniasis?. Pathog. Dis..

[17] Abbas A.K., Lichtman A.H., Pillai S. (2015). Cellular and Molecular Immunology.

[18] Bacellar O., Lessa H., Schriefer A., Machado P., Ribeiro de Jesus A., Dutra W.O., Gollob K.J., Carvalho E.M. (2002). Up-Regulation of Th1-Type Responses in Mucosal Leishmaniasis Patients. Infect. Immun..

[19] Toepp A.J., Petersen C.A. (2020). The Balancing Act: Immunology of Leishmaniosis. Res. Vet. Sci..

[20] Novais F.O., Carvalho L.P., Graff J.W., Beiting D.P., Ruthel G., Roos D.S., Betts M.R., Goldschmidt M.H., Wilson M.E., de Oliveira C.I. (2013). Cytotoxic T Cells Mediate Pathology and Metastasis in Cutaneous Leishmaniasis. PLoS Pathog..

[21] Rossi M., Fasel N. (2018). How to Master the Host Immune System? Leishmania Parasites Have the Solutions!. Int. Immunol..

[22] Miles S.A., Conrad S.M., Alves R.G., Jeronimo S.M.B., Mosser D.M. (2005). A Role for IgG Immune Complexes during Infection with the Intracellular Pathogen Leishmania. J. Exp. Med..

[23] Goncalves R., Christensen S.M., Mosser D.M. (2020). Humoral Immunity in Leishmaniasis—Prevention or Promotion of Parasite Growth?. Cytokine X.

[24] Costa C.H.N., Stewart J.M., Dissanayake S., Maguire J.H., Shaw J.J., David J.R., Bozza M., Ramos P.K.S., Silva M.R.B., Santos R.S. (2002). Asymptomatic Human Carriers of Leishmania Chagasi. Am. J. Trop. Med. Hyg..

[25] de Vries H.J.C., Schallig H.D. (2022). Cutaneous Leishmaniasis: A 2022 Updated Narrative Review into Diagnosis and Management Developments. Am. J. Clin. Dermatol..

[26] Kumar A., Pandey S.C., Samant M. (2020). A Spotlight on the Diagnostic Methods of a Fatal Disease Visceral Leishmaniasis. Parasite Immunol..

[27] Freire M.L., Machado de Assis T., Oliveira E., Moreira de Avelar D., Siqueira I.C., Barral A., Rabello A., Cota G. (2019). Performance of Serological Tests Available in Brazil for the Diagnosis of Human Visceral Leishmaniasis. PLoS Negl. Trop. Dis..

[28] Sato C.M., Sanchez M.C.A., Celeste B.J., Duthie M.S., Guderian J., Reed S.G., de Brito M.E.F., Campos M.B., de Souza Encarnação H.V., Guerra J. (2017). Use of Recombinant Antigens for Sensitive Serodiagnosis of American Tegumentary Leishmaniasis Caused by Different Leishmania Species. J. Clin. Microbiol..

[29] Link J.S., Alban S.M., Soccol C.R., Pereira G.V.M., Thomaz Soccol V. (2017). Synthetic Peptides as Potential Antigens for Cutaneous Leishmaniosis Diagnosis. J. Immunol. Res..

[30] Menezes-Souza D., Mendes T.A.d.O., Gomes M.d.S., Bartholomeu D.C., Fujiwara R.T. (2015). Improving serodiagnosis of human and canine leishmaniasis with recombinant Leishmania braziliensis cathepsin L-like protein and a synthetic peptide containing its linear B-cell epitope. PLoS Negl. Trop. Dis..

[31] Dias D.S., Ribeiro P.A., Salles B.C., Santos T.T., Ramos F.F., Lage D.P., Costa L.E., Portela A.S., Carvalho G.B., Chávez-Fumagalli M.A. (2018). Serological diagnosis and prognostic of tegumentary and visceral leishmaniasis using a conserved *Leishmania* hypothetical protein. Parasitol. Int..

[32] Rodrigues M.R., Santos L.M., Miyazaki C.K., Martins V.T., Ludolf F.R., Kursancew A.C., Ramos F.F., Dias D.S., Oliveira J.S., Vieira P.M. (2019). Immunodiagnosis of human and canine visceral leishmaniasis using recombinant *Leishmania infantum* Prohibitin protein and a synthetic peptide containing its conformational B-cell epitope. J. Immunol. Methods.

[33] Lima M.P., Costa L.E., Duarte M.C., Menezes-Souza D., Salles B.C.S., de Oliveira Santos T.T., Ramos F.F., Chávez-Fumagalli M.A., Kursancew A.C.S., Ambrosio R.P. (2017). Evaluation of a hypothetical protein for serodiagnosis and as a potential marker for post-treatment serological evaluation of tegumentary leishmaniasis patients. Parasitol. Res..

[34] de Souza L.M., Carvalho J., Bates M.D., Petterle R.R., Thomaz-Soccol V., Bates P.A. (2019). Production of a kinesin-related recombinant protein (Lbk39) from *Leishmania braziliensis* by *Leishmania tarentolae* promastigotes and its application in the serodiagnosis of leishmaniasis. One Health.

[35] Carvalho A.M., Costa L.E., Salles B.C., Santos T.T., Ramos F.F., Lima M.P., Chávez-Fumagalli M.A., Silvestre B.T., Portela Á.S., Roatt B.M. (2017). An ELISA immunoassay employing a conserved *Leishmania* hypothetical protein for the serodiagnosis of visceral and tegumentary leishmaniasis in dogs and humans. Cell. Immunol..

[36] Machado A.S., Ramos F.F., Oliveira-Da-Silva J.A., Santos T.T., Ludolf F., Tavares G.S., Costa L.E., Lage D.P., Steiner B.T., Chaves A.T. (2020). Leishmania infantum hypothetical protein evaluated as a recombinant protein and specific B-cell epitope for the serodiagnosis and prognosis of visceral leishmaniasis. Acta Trop..

[37] Galvani N.C., Machado A.S., Lage D.P., Freitas C.S., Vale D.L., de Oliveira D., Ludolf F., Ramos F.F., Fernandes B.B., Luiz G.P. (2021). ChimLeish, a new recombinant chimeric protein evaluated as a diagnostic and prognostic marker for visceral leishmaniasis and human immunodeficiency virus coinfection. Parasitol. Res..

[38] Salles B.C., Dias D.S., Steiner B.T., Lage D.P., Ramos F.F., Ribeiro P.A., Santos T.T., Lima M.P., Costa L.E., Chaves A.T. (2019). Potential application of small myristoylated protein-3 evaluated as recombinant antigen and a synthetic peptide containing its linear B-cell epitope for the serodiagnosis of canine visceral and human tegumentary leishmaniasis. Immunobiology.

[39] Santos T.T., Cardoso M.S., Machado A.S., Siqueira W.F., Ramos F.F., Oliveira-da-Silva J.A., Tavares G.S., Lage D.P., Costa L.E., de Freitas C.S. (2019). Recombinant *Leishmania* eukaryotic elongation factor-1 beta protein: A potential diagnostic antigen to detect tegumentary and visceral leishmaniasis in dogs and humans. Microb. Pathog..

[40] Dhom-Lemos L., Viana A.G., Cunha J.L.R., Cardoso M.S., Mendes T.A.O., Pinheiro G.R.G., Siqueira W.F., Lobo F.P., Teles L.F., Bueno L.L. (2019). Leishmania Infantum Recombinant Kinesin Degenerated Derived Repeat (RKDDR): A Novel Potential Antigen for Serodiagnosis of Visceral Leishmaniasis. PLoS ONE.

[41] Ghosh P., Bhaskar K.R., Hossain F., Khan M.A., Vallur A.C., Duthie M.S., Hamano S., Salam M.A., Huda M.M., Khan M.G. (2016). Evaluation of diagnostic performance of rK28 ELISA using urine for diagnosis of visceral leishmaniasis. Parasites Vectors.

[42] Martins V.T., Duarte M.C., Chávez-Fumagalli M.A., Menezes-Souza D., Coelho C.S., de Magalhães-Soares D.F., Fernandes A.P., Soto M., Tavares C.A., Coelho E.A. (2015). A Leishmania-specific hypothetical protein expressed in both promastigote and amastigote stages of Leishmania infantum employed for the serodiagnosis of, and as a vaccine candidate against, visceral leishmaniasis. Parasites Vectors.

[43] Regenmortel M.H.V. (2009). What Is a B-Cell Epitope?. Epitope Mapping Protocols.

[44] Flower D.R., Flower D.R. (2007). Immunoinformatics.

[45] Ponomarenko J., Van Regenmortel M. (2009). B Cell Epitope Prediction. Struct. Bioinform..

[46] Yao B., Zhang L., Liang S., Zhang C. (2012). SVMTriP: A Method to Predict Antigenic Epitopes Using Support Vector Machine to Integrate Tri-Peptide Similarity and Propensity. PLoS ONE.

[47] Hopp T.P., Woods K.R. (1981). Prediction of Protein Antigenic Determinants from Amino Acid Sequences. Proc. Natl. Acad. Sci. USA.

[48] Saha S., Raghava G.P.S. (2004). BcePred: Prediction of Continuous B-Cell Epitopes in Antigenic Sequences Using Physico-Chemical Properties. International Conference on Artificial Immune Systems.

[49] Saha S., Raghava G.P.S. (2006). Prediction of Continuous B-cell Epitopes in an Antigen Using Recurrent Neural Network. Proteins Struct. Funct. Bioinform..

[50] Singh H., Ansari H.R., Raghava G.P.S. (2013). Improved Method for Linear B-Cell Epitope Prediction Using Antigen’s Primary Sequence. PLoS ONE.

[51] Clifford J.N., Høie M.H., Deleuran S., Peters B., Nielsen M., Marcatili P. (2022). BepiPred-3.0: Improved B-cell Epitope Prediction Using Protein Language Models. Protein Sci..

[52] Kulkarni-Kale U., Bhosle S., Kolaskar A.S. (2005). CEP: A Conformational Epitope Prediction Server. Nucleic Acids Res..

[53] Haste Andersen P., Nielsen M., Lund O. (2006). Prediction of Residues in Discontinuous B-cell Epitopes Using Protein 3D Structures. Protein Sci..

[54] Ponomarenko J., Bui H.-H., Li W., Fusseder N., Bourne P.E., Sette A., Peters B. (2008). ElliPro: A New Structure-Based Tool for the Prediction of Antibody Epitopes. BMC Bioinform..

[55] Sun J., Wu D., Xu T., Wang X., Xu X., Tao L., Li Y.X., Cao Z.W. (2009). SEPPA: A Computational Server for Spatial Epitope Prediction of Protein Antigens. Nucleic Acids Res..

[56] Rubinstein N.D., Mayrose I., Martz E., Pupko T. (2009). Epitopia: A Web-Server for Predicting B-Cell Epitopes. BMC Bioinform..

[57] Ansari H., Raghava G.P. (2010). Identification of Conformational B-Cell Epitopes in an Antigen from Its Primary Sequence. Immunome Res..

[58] Negi S.S., Braun W. (2009). Automated Detection of Conformational Epitopes Using Phage Display Peptide Sequences. Bioinform. Biol. Insights.

[59] Moreau V., Granier C., Villard S., Laune D., Molina F. (2006). Discontinuous Epitope Prediction Based on Mimotope Analysis. Bioinformatics.

[60] Qi Y., Zheng P., Huang G. (2023). DeepLBCEPred: A Bi-LSTM and Multi-Scale CNN-Based Deep Learning Method for Predicting Linear B-Cell Epitopes. Front. Microbiol..

[61] Zeng Y., Wei Z., Yuan Q., Chen S., Yu W., Lu Y., Gao J., Yang Y. (2023). Identifying B-Cell Epitopes Using AlphaFold2 Predicted Structures and Pretrained Language Model. Bioinformatics.

[62] Ivanisenko N.V., Shashkova T.I., Shevtsov A., Sindeeva M., Umerenkov D., Kardymon O. (2024). SEMA 2.0: Web-Platform for B-Cell Conformational Epitopes Prediction Using Artificial Intelligence. Nucleic Acids Res..

[63] Hopp T.P., Woods K.R. (1983). A Computer Program for Predicting Protein Antigenic Determinants. Mol. Immunol..

[64] EL-Manzalawy Y., Honavar V. (2010). Recent Advances in B-Cell Epitope Prediction Methods. Immunome Res..

[65] Wilkins M.R., Gasteiger E., Bairoch A., Sanchez J.-C., Williams K.L., Appel R.D., Hochstrasser D.F. (2005). Protein Identification and Analysis Tools in the ExPASy Server. 2-D Proteome Analysis Protocols.

[66] Odorico M., Pellequer J. (2003). BEPITOPE: Predicting the Location of Continuous Epitopes and Patterns in Proteins. J. Mol. Recognit..

[67] Galvani N.C., Machado A.S., Lage D.P., Martins V.T., de Oliveira D., Freitas C.S., Vale D.L., Fernandes B.B., Oliveira-da-Silva J.A., Reis T.A.R. (2022). Sensitive and Specific Serodiagnosis of Tegumentary Leishmaniasis Using a New Chimeric Protein Based on Specific B-Cell Epitopes of Leishmania Antigenic Proteins. Microb. Pathog..

[68] Jespersen M.C., Peters B., Nielsen M., Marcatili P. (2017). BepiPred-2.0: Improving Sequence-Based B-Cell Epitope Prediction Using Conformational Epitopes. Nucleic Acids Res..

[69] Lin Z., Akin H., Rao R., Hie B., Zhu Z., Lu W., Smetanin N., Verkuil R., Kabeli O., Shmueli Y. (2023). Evolutionary-Scale Prediction of Atomic-Level Protein Structure with a Language Model. Science.

[70] Moreira G., Maia R., Soares N., Ostolin T., Coura-Vital W., Aguiar-Soares R., Ruiz J., Resende D., de Brito R., Reis A. (2024). Synthetic Peptides Selected by Immunoinformatics as Potential Tools for the Specific Diagnosis of Canine Visceral Leishmaniasis. Microorganisms.

[71] Medeiros R.M.T.E., Carvalho A.M.R.S., Ferraz I.d.A., Medeiros F.A.C., Cruz L.d.R., Rocha M.O.d.C., Coelho E.A.F., Gonçalves D.U., Mendes T.A.d.O., Duarte M.C. (2022). Mapping Linear B-Cell Epitopes of the Tryparedoxin Peroxidase and Its Implications in the Serological Diagnosis of Tegumentary Leishmaniasis. Acta Trop..

[72] Sanchez-Trincado J.L., Gomez-Perosanz M., Reche P.A. (2017). Fundamentals and Methods for T- and B-Cell Epitope Prediction. J. Immunol. Res..

[73] Thornton J.M., Edwards M.S., Taylor W.R., Barlow D.J. (1986). Location of ‘Continuous’ Antigenic Determinants in the Protruding Regions of Proteins. EMBO J..

[74] Eswar N., Webb B., Marti-Renom M.A., Madhusudhan M.S., Eramian D., Shen M., Pieper U., Sali A. (2006). Comparative Protein Structure Modeling Using Modeller. Curr. Protoc. Bioinform..

[75] Hanson R.M. (2010). Jmol—A Paradigm Shift in Crystallographic Visualization. J. Appl. Crystallogr..

[76] Costa S.S., Santos L.M.O., Freire L.C., Tedeschi A.L.F., Ribeiro N.R., Queiroz M.H.R., Neto E.B., Pimenta D.C., Galvani N.C., Luiz G.P. (2023). Immunoproteomics Approach for the Discovery of Antigens Applied to the Diagnosis of Canine Visceral Leishmaniasis. Acta Trop..

[77] Arab-Mazar Z., Mohebali M., Ranjbar M.M., Tabaei S.J.S., Mamaghani A.J., Taghipour N. (2022). Design of a Polytopic Construct of LACK, TSA and GP63 Proteins for the Diagnosis of Cutaneous Leishmaniasis: An in Silico Strategy. J. Asia Pac. Entomol..

[78] Assis L.M., Sousa J.R., Pinto N.F.S., Silva A.A., Vaz A.F.M., Andrade P.P., Carvalho E.M., De Melo M.A. (2014). B-cell Epitopes of Antigenic Proteins in *Leishmania infantum*: An in Silico Analysis. Parasite Immunol..

[79] Geysen H.M., Rodda S.J., Mason T.J. (1986). A Priori Delineation of a Peptide Which Mimics a Discontinuous Antigenic Determinant. Mol. Immunol..

[80] LeCun Y., Bengio Y., Hinton G. (2015). Deep Learning. Nature.

[81] Jiang H., Li S., Liu W., Zheng H., Liu J., Zhang Y. (2020). Geometry-Aware Cell Detection with Deep Learning. mSystems.

[82] Li S., Yang Q., Jiang H., Cortés-Vecino J.A., Zhang Y. (2020). Parasitologist-Level Classification of Apicomplexan Parasites and Host Cell with Deep Cycle Transfer Learning (DCTL). Bioinformatics.

[83] Quan Q., Wang J., Liu L. (2020). An Effective Convolutional Neural Network for Classifying Red Blood Cells in Malaria Diseases. Interdiscip. Sci..

[84] Kassim Y.M., Palaniappan K., Yang F., Poostchi M., Palaniappan N., Maude R.J., Antani S., Jaeger S. (2021). Clustering-Based Dual Deep Learning Architecture for Detecting Red Blood Cells in Malaria Diagnostic Smears. IEEE J. Biomed. Health Inform..

[85] Ren S., He K., Girshick R., Sun J. (2017). Faster R-CNN: Towards Real-Time Object Detection with Region Proposal Networks. IEEE Trans. Pattern Anal. Mach. Intell..

[86] Shelhamer E., Long J., Darrell T. (2017). Fully Convolutional Networks for Semantic Segmentation. IEEE Trans. Pattern Anal. Mach. Intell..

[87] Milletari F., Navab N., Ahmadi S.-A. V-Net: Fully Convolutional Neural Networks for Volumetric Medical Image Segmentation. Proceedings of the 2016 Fourth International Conference on 3D Vision (3DV).

[88] Krizhevsky A., Sutskever I., Hinton G.E. (2017). ImageNet Classification with Deep Convolutional Neural Networks. Commun. ACM.

[89] Górriz M., Aparicio A., Raventós B., Vilaplana V., Sayrol E., López-Codina D. (2018). Leishmaniasis Parasite Segmentation and Classification Using Deep Learning. Articulated Motion and Deformable Objects: 10th International Conference, AMDO 2018, Palma de Mallorca, Spain, 12–13 July 2018.

[90] Arce-Lopera C.A., Diaz-Cely J., Quintero L. (2021). Presumptive Diagnosis of Cutaneous Leishmaniasis. Front. Health Inform..

[91] Li H., Soto-Montoya H., Voisin M., Valenzuela L.F., Prakash M. (2019). Octopi: Open configurable high-throughput imaging platform for infectious disease diagnosis in the field. BioRxiv.

[92] Gonçalves C., Borges A., Dias V., Andrade N., Aguiar B., Silva R. Método Automático Para Detecção de Leishmaniose Visceral Em Humanos. Proceedings of the Congresso Brasileiro de Automática—CBA.

[93] Zare M., Akbarialiabad H., Parsaei H., Asgari Q., Alinejad A., Bahreini M.S., Hosseini S.H., Ghofrani-Jahromi M., Shahriarirad R., Amirmoezzi Y. (2022). A Machine Learning-Based System for Detecting Leishmaniasis in Microscopic Images. BMC Infect. Dis..

[94] Leal J.F.d.C., Barroso D.H., Trindade N.S., de Miranda V.L., Gurgel-Gonçalves R. (2023). Automated Identification of Cutaneous Leishmaniasis Lesions Using Deep-Learning-Based Artificial Intelligence. Biomedicines.

[95] de Souza E.P., Gomes C.M., Barroso D.H., de Miranda V.L., Gurgel-Gonçalves R. (2019). Aplicações Do Deep Learning Para Diagnóstico de Doenças e Identificação de Insetos Vetores. Saúde Em Debate.

[96] Zhang C., Jiang H., Liu W., Li J., Tang S., Juhas M., Zhang Y. (2022). Correction of Out-of-Focus Microscopic Images by Deep Learning. Comput. Struct. Biotechnol. J..

[97] Huang B., Babcock H., Zhuang X. (2010). Breaking the Diffraction Barrier: Super-Resolution Imaging of Cells. Cell.

[98] Jumper J., Evans R., Pritzel A., Green T., Figurnov M., Ronneberger O., Tunyasuvunakool K., Bates R., Žídek A., Potapenko A. (2021). Highly Accurate Protein Structure Prediction with AlphaFold. Nature.

[99] Raybould M.I.J., Marks C., Lewis A.P., Shi J., Bujotzek A., Taddese B., Deane C.M. (2020). Thera-SAbDab: The Therapeutic Structural Antibody Database. Nucleic Acids Res..

[100] Poiron C., Wu Y., Ginestoux C., Ehrenmann F., Duroux P., Lefranc M. (2010). PIMGT/mAb-DB: The IMGT® database for therapeutic monoclonal antibodies. Informatique et Mathématiques (JOBIM), Montpellier. Nucleic Acids Res..

[101] Roy A., Nair S., Sen N., Soni N., Madhusudhan M.S. (2017). In Silico Methods for Design of Biological Therapeutics. Methods.

[102] Sela-Culang I., Benhnia M.R.-E., Matho M.H., Kaever T., Maybeno M., Schlossman A., Nimrod G., Li S., Xiang Y., Zajonc D. (2014). Using a Combined Computational-Experimental Approach to Predict Antibody-Specific B Cell Epitopes. Structure.

[103] Chen R., Li L., Weng Z. (2003). ZDOCK: An Initial-stage Protein-docking Algorithm. Proteins Struct. Funct. Bioinform..

[104] Schneidman-Duhovny D., Inbar Y., Nussinov R., Wolfson H.J. (2005). PatchDock and SymmDock: Servers for Rigid and Symmetric Docking. Nucleic Acids Res..

[105] Sircar A., Gray J.J. (2010). SnugDock: Paratope Structural Optimization during Antibody-Antigen Docking Compensates for Errors in Antibody Homology Models. PLoS Comput. Biol..

[106] Ramírez-Aportela E., López-Blanco J.R., Chacón P. (2016). FRODOCK 2.0: Fast Protein–Protein Docking Server. Bioinformatics.

[107] van Zundert G.C.P., Rodrigues J.P.G.L.M., Trellet M., Schmitz C., Kastritis P.L., Karaca E., Melquiond A.S.J., van Dijk M., de Vries S.J., Bonvin A.M.J.J. (2016). The HADDOCK2.2 Web Server: User-Friendly Integrative Modeling of Biomolecular Complexes. J. Mol. Biol..

[108] Kozakov D., Hall D.R., Xia B., Porter K.A., Padhorny D., Yueh C., Beglov D., Vajda S. (2017). The ClusPro Web Server for Protein–Protein Docking. Nat. Protoc..

[109] Jeliazkov J.R., Frick R., Zhou J., Gray J.J. (2021). Robustification of RosettaAntibody and Rosetta SnugDock. PLoS ONE.

[110] Méndez R., Leplae R., De Maria L., Wodak S.J. (2003). Assessment of Blind Predictions of Protein–Protein Interactions: Current Status of Docking Methods. Proteins Struct. Funct. Bioinform..

[111] Guest J.D., Vreven T., Zhou J., Moal I., Jeliazkov J.R., Gray J.J., Weng Z., Pierce B.G. (2021). An Expanded Benchmark for Antibody-Antigen Docking and Affinity Prediction Reveals Insights into Antibody Recognition Determinants. Structure.

[112] Lari A., Lari N., Biabangard A. (2022). Immunoinformatics Approach to Design a Novel Subunit Vaccine Against Visceral Leishmaniasis. Int. J. Pept. Res. Ther..

[113] Khan A.A., Ami J.Q., Faisal K., Chowdhury R., Ghosh P., Hossain F., El Wahed A.A., Mondal D. (2020). An Immunoinformatic Approach Driven by Experimental Proteomics: In Silico Design of a Subunit Candidate Vaccine Targeting Secretory Proteins of Leishmania Donovani Amastigotes. Parasit. Vectors.

[114] Shams M., Nourmohammadi H., Majidiani H., Shariatzadeh S.A., Asghari A., Fatollahzadeh M., Irannejad H. (2022). Engineering a Multi-Epitope Vaccine Candidate against Leishmania Infantum Using Comprehensive Immunoinformatics Methods. Biologia.

[115] Onile O.S., Musaigwa F., Ayawei N., Omoboyede V., Onile T.A., Oghenevovwero E., Aruleba R.T. (2022). Immunoinformatics Studies and Design of a Potential Multi-Epitope Peptide Vaccine to Combat the Fatal Visceral Leishmaniasis. Vaccines.

[116] Saha S., Vashishtha S., Kundu B., Ghosh M. (2022). In-Silico Design of an Immunoinformatics Based Multi-Epitope Vaccine against Leishmania Donovani. BMC Bioinform..

[117] Khatoon N., Pandey R.K., Prajapati V.K. (2017). Exploring Leishmania Secretory Proteins to Design B and T Cell Multi-Epitope Subunit Vaccine Using Immunoinformatics Approach. Sci. Rep..

[118] Silva R.d.F.e., Ferreira L.F.G.R., Hernandes M.Z., de Brito M.E.F., de Oliveira B.C., da Silva A.A., De-Melo-Neto O.P., Rezende A.M., Pereira V.R.A. (2016). Combination of In Silico Methods in the Search for Potential CD4+ and CD8+ T Cell Epitopes in the Proteome of Leishmania Braziliensis. Front. Immunol..

[119] Tavares D.H.C. (2018). Evaluation of Flow Cytometry in the Diagnosis of Leishmaniasis Using Recombinant Antigens. Doctoral Thesis.

[120] Jamal F., Dikhit M.R., Singh M.K., Shivam P., Kumari S., Pushpanjali S., Dubey A.K., Kumar P., Narayan S., Gupta A.K. (2017). Identification of B-cell epitope of Leishmania donovani and its application in diagnosis of visceral leishmaniasis. J. Biomol. Struct. Dyn..

[121] Yaghoubi P., Bandehpour M., Mohebali M., Akhoundi B., Kazemi B. (2021). Designing and evaluation of a recombinant multiepitope protein by using ELISA for diagnosis of Leishmania infantum infection in dogs. Iran. J. Parasitol..

[122] Siqueira W.F., Cardoso M.S., Clímaco M.d.C., Silva A.L.T., Heidt B., Eersels K., van Grinsven B., Bartholomeu D.C., Bueno L.L., Cleij T. (2023). Serodiagnosis of leishmaniasis in asymptomatic and symptomatic dogs by use of the recombinant dynamin-1-like protein from Leishmania infantum: A preliminary study. Acta Trop..

[123] Hashemzadeh P., Bandehpour M., Kheirandish F., Dariushnejad H., Mohamadi M., Rouzbahani A.K. Design and evaluation of a novel multi-epitope antigen for evaluating the diagnostic immune responses against Leishmania infantum infection. Preprint.

[124] Lima B., Pires S., Fialho L., Oliveira E., Machado-De-Avila R., Chávez-Olórtegui C., Chapeaurouge A., Perales J., Andrade H. (2017). A proteomic road to acquire an accurate serological diagnosis for human tegumentary leishmaniasis. J. Proteom..

[125] Fonseca T., Faria A., Leite H., da Silveira J., Carneiro C., Andrade H. (2019). Chemiluminescent ELISA with multi-epitope proteins to improve the diagnosis of canine visceral leishmaniasis. Vet. J..

[126] Faria A.R., Veloso L.d.C., Coura-Vital W., Reis A.B., Damasceno L.M., Gazzinelli R.T., Andrade H.M. (2015). Novel recombinant multiepitope proteins for the diagnosis of asymptomatic Leishmania infantum-infected dogs. PLoS Negl. Trop. Dis..

[127] Volpe J., Parchen G.P., Costa F.S., de Souza Silva A., Andrade H.M., Amaral C.D., Silva S.M., Kubota L.T., Souto D.E. (2024). Synthetic peptides-based SPR biosensor evaluation towards canine visceral leishmaniasis diagnosis: A simple and effective approach. Microchem. J..

[128] Vale D.L., Dias D.S., Machado A.S., Ribeiro P.A., Lage D.P., Costa L.E., Steiner B.T., Tavares G.S., Ramos F.F., Martínez-Rodrigo A. (2019). Diagnostic evaluation of the amastin protein from Leishmania infantum in canine and human visceral leishmaniasis and immunogenicity in human cells derived from patients and healthy controls. Diagn. Microbiol. Infect. Dis..

[129] Lage D.P., Martins V.T., Duarte M.C., Costa L.E., Garde E., Dimer L.M., Coelho E.A. (2016). A new Leishmania-specific hypothetical protein and its non-described specific B cell conformational epitope applied in the serodiagnosis of canine visceral leishmaniasis. Parasitol. Res..

[130] Neto S.Y., Souto D.E.P., de Andrade H.M., Luz R.d.C.S., Kubota L.T., Damos F.S. (2018). Visible LED light driven photoelectroanalytical detection of antibodies of visceral leishmaniasis based on electrodeposited CdS film sensitized with Au nanoparticles. Sens. Actuators B Chem..

[131] Hinckel B.C.B., Marlais T., Airs S., Bhattacharyya T., Imamura H., Dujardin J.-C., El-Safi S., Singh O.P., Sundar S., Falconar A.K. (2019). Refining wet lab experiments with in silico searches: A rational quest for diagnostic peptides in visceral leishmaniasis. PLoS Negl. Trop. Dis..

[132] Farooq U. (2019). Epitope prediction and structural analysis of sterol 24-c-methyltransferase antigen of Leishmania donovani using in silico approach. EC Microbiol..

[133] Rath K. (2016). Mapping B-Cell Epitopes for the Hypothetical Proteins of Leishmania Donovani and Its Potential for the Clinical Diagnosis of Visceral Leishmaniasis. Ph.D. Thesis.

[134] Carvalho A.M.R.S., Mendes T.A.d.O., Coelho E.A.F., Duarte M.C., Menezes-Souza D. (2018). New antigens for the serological diagnosis of human visceral leishmaniasis identified by immunogenomic screening. PLoS ONE.

[135] Steffler J.M.D. (2023). Identificação e Clonagem de Genes Espécie-Específicos de Leishmania Infantum Para Expressão Piloto de Proteínas Recombinantes com Potencial Para o Diagnóstico de Leishmaniose Visceral. Ph.D. Thesis.

[136] Menezes-Souza D., Mendes T.A.d.O., Nagem R.A.P., Santos T.T.d.O., Silva A.L.T., Santoro M.M., de Carvalho S.F.G., Coelho E.A.F., Bartholomeu D.C., Fujiwara R.T. (2014). Mapping B-cell epitopes for the peroxidoxin of Leishmania (Viannia) braziliensis and its potential for the clinical diagnosis of tegumentary and visceral leishmaniasis. PLoS ONE.

[137] Siqueira W.F., Viana A.G., Cunha J.L.R., Rosa L.M., Bueno L.L., Bartholomeu D.C., Cardoso M.S., Fujiwara R.T. (2021). The increased presence of repetitive motifs in the KDDR-plus recombinant protein, a kinesin-derived antigen from Leishmania infantum, improves the diagnostic performance of serological tests for human and canine visceral leishmaniasis. PLoS Negl. Trop. Dis..

[138] Jesus M.S.D., Farias L.P., Carvalho F.M.L., Santos L.A.D., Passos N.M., Silva H.R.F.D., Neto O.P.D.M., Brodskyn C.I., Fraga D.B.M. (2024). Identifying Linear B-Cell Epitopes in Leishmania Infantum Recombinant Proteins Using Microarray Technology for Enhanced Serodiagnosis of Visceral Leishmaniasis.

[139] Menezes-Souza D., Mendes T.A.D.O., Gomes M.D.S., Reis-Cunha J.L., Nagem R.A.P., Carneiro C.M., Coelho E.A.F., Galvão L.M.D.C., Fujiwara R.T., Bartholomeu D.C. (2014). Epitope mapping of the HSP83.1 protein of Leishmania braziliensis discloses novel targets for immunodiagnosis of tegumentary and visceral clinical forms of leishmaniasis. Clin. Vaccine Immunol..

[140] Ejazi S.A., Bhattacharyya A., Choudhury S.T., Ghosh S., Sabur A., Pandey K., Das V.N.R., Das P., Rahaman M., Goswami R.P. (2018). Immunoproteomic identification and characterization of Leishmania membrane proteins as non-invasive diagnostic candidates for clinical visceral leishmaniasis. Sci. Rep..

[141] Mahdavi R., Shams-Eldin H., Witt S., Latz A., Heinz D., Fresco-Taboada A., Aira C., Hübner M.P., Sukyte D., Visekruna A. (2023). Development of a novel enzyme-linked immunosorbent assay and lateral flow test system for improved serodiagnosis of visceral leishmaniasis in different areas of endemicity. Microbiol. Spectr..

[142] Maia R.C. (2021). Seleção de Potenciais Peptídeos Para o Diagnóstico Sorológico da Leishmaniose Visceral Canina por Imunoinformática. Ph.D. Thesis.

[143] Teixeira H.C., Valle G.P.C., Mahdavi R., Dias P.S.M., De Oliveira E.E., Aira C., Heinz D., Latz A., de Lana M., Morgado F. (2024). Refinement of the rKLi8.3-based serodiagnostic ELISA allows detection of canine visceral leishmaniasis in dogs with low antibody titers. Pathogens.

[144] Marlais T., Bhattacharyya T., Pearson C., Gardner B.L., Marhoon S., Airs S., Hayes K., Falconar A.K., Singh O.P., Reed S.G. (2020). Isolation and characterization of Leishmania donovani protein antigens from urine of visceral leishmaniasis patients. PLoS ONE.

