# Supplementary material for: Exploring Bioinformatics Solutions for Improved Leishmaniasis Diagnostic Tools: A Review
Source: Molecules. 2024 Nov 7;29(22):5259. doi: 10.3390/molecules29225259 (PMC11596704; doi:10.3390/molecules29225259)
Supplement: Supplementary file 1 [file molecules-29-05259-s001.zip › molecules-3132986-supplementary.pdf]

Supplementary Material

Figure S1. Most frequently employed *in silico* tools in the past decade for the selection of novel diagnostic molecules for Leishmaniasis.

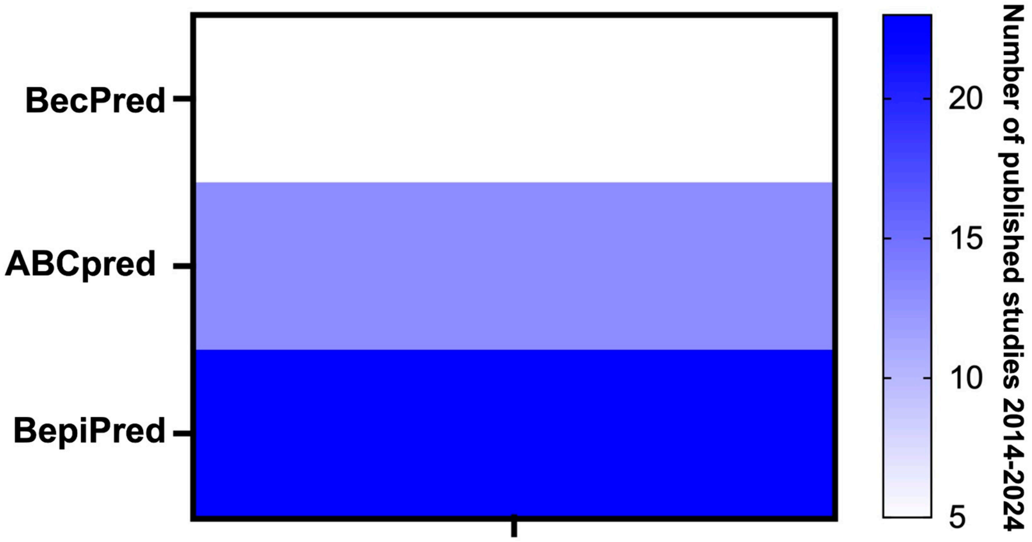

Legend: This figure presents the distribution of published studies from 2014 to 2024, classified according to three B-cell epitope prediction tools: BecPred, ABCpred, and BepiPred. The color gradient on the right represents the number of studies, ranging from 5 to 20. The results demonstrate that BepiPred has the highest frequency of publications, followed by ABCpred, while BecPred has the lowest number of studies published within the given timeframe. **Note:** References for the works included and cited in the figure are provided in the supplementary material.

References:

Tavares DHC. Evaluation of flow cytometry in the diagnosis of leishmaniasis using recombinant antigens [Doctoral thesis]. Recife: Instituto Aggeu Magalhães, Biosciences and Biotechnology in Health; 2018. 114 p.

Jamal F, Ahmad W, Arish M, et al. Identification of B-cell epitope of *Leishmania donovani* and its application in diagnosis of visceral leishmaniasis. J Biomol Struct Dyn. 2017;35(16):3569-3580. doi.org/10.1080/07391102.2016.1263240

Yaghoubi P, Marashifard M, Shirian S, et al. Designing and evaluation of a recombinant multiepitope protein by using ELISA for diagnosis of *Leishmania infantum* infection in dogs. Iran J Parasitol. 2021;16(3):377.doi: 10.18502/ijpa.v16i3.7090

Siqueira WF, Barbosa GM, Guimarães RM, et al. Serodiagnosis of leishmaniasis in asymptomatic and symptomatic dogs by use of the recombinant dynamin-1-like protein from *Leishmania infantum*: A preliminary study. Acta Trop. 2023;239:106827.doi.org/10.1016/j.actatropica.2023.106827

Moreira G, Souza AP, Lima D, et al. Synthetic peptides selected by immunoinformatics as potential tools for the specific diagnosis of canine visceral leishmaniasis. Microorganisms. 2024;12(5):906. doi.org/10.3390/microorganisms12050906

Hashemzadeh P, Bahonar A, Ghaffarifar F, et al. Design and evaluation of a novel multi-epitope antigen for evaluating the diagnostic immune responses against *Leishmania infantum* infection. 2024.https://doi.org/10.21203/rs.3.rs-4143767/v1

Jamal F, Ahmad W, Arish M, et al. Identification of B-cell epitope of *Leishmania donovani* and its application in diagnosis of visceral leishmaniasis. J Biomol Struct Dyn. 2017;35(16):3569-3580.doi.org/10.1080/07391102.2016.1263240

Lima BSS, Coelho VTS, Rugani JN, et al. A proteomic road to acquire an accurate serological diagnosis for human tegumentary leishmaniasis. J Proteomics. 2017;151:174-181.doi.org/10.1016/j.jprot.2016.05.017

Fonseca THS, Fukutani KF, Silva LS, et al. Chemiluminescent ELISA with multi-epitope proteins to improve the diagnosis of canine visceral leishmaniasis. Vet J. 2019;253:105387.doi.org/10.1016/j.tvjl.2019.105387

Yaghoubi P, Marashifard M, Shirian S, et al. Designing and evaluation of a recombinant multiepitope protein by using ELISA for diagnosis of *Leishmania infantum* infection in dogs. Iran J Parasitol. 2021;16(3):377.doi: 10.18502/ijpa.v16i3.7090

Faria AR, Reis AB, Lima AP, et al. Novel recombinant multiepitope proteins for the diagnosis of asymptomatic *Leishmania infantum*-infected dogs. PLoS Negl Trop Dis. 2015;9(1):e3429. doi.org/10.1371/journal.pntd.0003429

Volpe J, Tonhosolo R, Toma HK, et al. Synthetic peptides-based SPR biosensor evaluation towards canine visceral leishmaniasis diagnosis: A simple and effective approach. Microchem J. 2024;110844.doi.org/10.1016/j.microc.2024.110844

Vale DL, Faria AR, Reis AB, et al. Diagnostic evaluation of the amastin protein from *Leishmania infantum* in canine and human visceral leishmaniasis and immunogenicity in human cells derived from patients and healthy controls. Diagn Microbiol Infect Dis. 2019;95(2):134-143.doi.org/10.1016/j.diagmicrobio.2019.04.015

Lage DP, Mendonça LZ, Chávez-Fumagalli MA, et al. A new *Leishmania*-specific hypothetical protein and its non-described specific B cell conformational epitope applied in the serodiagnosis of canine visceral leishmaniasis. Parasitol Res. 2016;115:1649-1658.doi.org/10.1007/s00436-016-4904-x

Machado AS, Lage DP, Mendonça LZ, et al. *Leishmania infantum* hypothetical protein evaluated as a recombinant protein and specific B-cell epitope for the serodiagnosis and prognosis of visceral leishmaniasis. Acta Trop. 2020;203:105318.doi.org/10.1016/j.actatropica.2019.105318

Neto SY, Castro LF, Santana ER, et al. Visible LED light driven photoelectroanalytical detection of antibodies of visceral leishmaniasis based on electrodeposited CdS film sensitized with Au nanoparticles. Sens Actuators B Chem. 2018;256:682-690.doi.org/10.1016/j.snb.2017.09.202

Bremer Hinckel BC, de Rezende AM, Lima BS, et al. Refining wet lab experiments with in silico searches: A rational quest for diagnostic peptides in visceral leishmaniasis. PLoS Negl Trop Dis. 2019;13(5).doi.org/10.1371/journal.pntd.0007353

Farooq U, Shakeel S, Shahid S, et al. Epitope prediction and structural analysis of sterol 24-c-methyltransferase antigen of *Leishmania donovani* using in silico approach. EC Microbiol. 2019;15:691-699.

Rath K. Mapping B-cell epitopes for the hypothetical proteins of *Leishmania donovani* and its potential for the clinical diagnosis of visceral leishmaniasis [Doctoral thesis]. 2016.

Carvalho AMRS, Luz NF, Silva MV, et al. New antigens for the serological diagnosis of human visceral leishmaniasis identified by immunogenomic screening. PLoS One. 2018;13(12).https://doi.org/10.1371/journal.pone.0209599

Moreira G, Souza AP, Lima D, et al. Synthetic peptides selected by immunoinformatics as potential tools for the specific diagnosis of canine visceral leishmaniasis. Microorganisms. 2024;12(5):906.https://doi.org/10.3390/microorganisms12050906

Dhom-Lemos L, Lima AP, Gontijo NF, et al. *Leishmania infantum* recombinant kinesin degenerated derived repeat (rKDDR): A novel potential antigen for serodiagnosis of visceral leishmaniasis. PLoS One. 2019;14(1).https://doi.org/10.1371/journal.pone.0211719

Steffler JMD. Identificação e clonagem de genes espécie-específicos de *Leishmania infantum* para expressão piloto de proteínas recombinantes com potencial para o diagnóstico de Leishmaniose Visceral [Tese de Doutorado]. 2023.

Menezes-Souza D, Guimarães ET, Ramos FF, et al. Improving serodiagnosis of human and canine leishmaniasis with recombinant *Leishmania braziliensis* cathepsin L-like protein and a synthetic peptide containing its linear B-cell epitope. PLoS Negl Trop Dis. 2015;9(1).doi.org/10.1371/journal.pntd.0003426

Menezes-Souza D, Mendes O, Pinto Nagem RA, Santos O, Teixeira Silva AL, Santoro MM, et al. Mapping B-cell epitopes for the peroxidoxin of *Leishmania (Viannia) braziliensis* and its potential for the clinical diagnosis of tegumentary and visceral leishmaniasis. PLoS One. 2014;9(6).doi.org/10.1371/journal.pone.0099216

Siqueira WF, Barbosa GM, Guimarães RM, et al. Serodiagnosis of leishmaniasis in asymptomatic and symptomatic dogs by use of the recombinant dynamin-1-like protein from *Leishmania infantum*: A preliminary study. Acta Trop. 2023;239:106827.doi.org/10.1016/j.actatropica.2023.106827

Menezes-Souza D, Guimarães ET, Ramos FF, et al. Linear B-cell epitope mapping of MAPK3 and MAPK4 from *Leishmania braziliensis*: Implications for the serodiagnosis of human and canine leishmaniasis. Appl Microbiol Biotechnol.2015;99:doi.org/10.1007/s00253-014-6168-7

Yaghoubi P, Marashifard M, Shirian S, et al. Designing and evaluation of a recombinant multiepitope protein by using ELISA for diagnosis of *Leishmania infantum* infection in dogs. Iran J Parasitol. 2021;16(3):377.doi: 10.18502/ijpa.v16i3.7090

Siqueira WF, Jesus MS, Lima BS, et al. The increased presence of repetitive motifs in the KDDR-plus recombinant protein, a kinesin-derived antigen from *Leishmania infantum*, improves the diagnostic performance of serological tests for human and canine visceral leishmaniasis. PLoS Negl Trop Dis. 2021;15(9).doi.org/10.1371/journal.pntd.0009759

Medeiros RMTE, Pinto MD, dos Santos SA, et al. Mapping linear B-cell epitopes of the tryparedoxin peroxidase and its implications in the serological diagnosis of tegumentary leishmaniasis. Acta Trop. 2022;232:doi.org/10.1016/j.actatropica.2022.106521

Jesus MS, Guimarães AP, Carvalho E, et al. Identifying linear B-cell epitopes in *Leishmania infantum* recombinant proteins using microarray technology for enhanced serodiagnosis of visceral leishmaniasis. 2024.

Menezes-Souza D, Mendes O, Guimarães ET, et al. Epitope mapping of the HSP83.1 protein of *Leishmania braziliensis* discloses novel targets for immunodiagnosis of tegumentary and visceral clinical forms of leishmaniasis. Clin Vaccine Immunol. 2014;21(7):949-959.

Ejazi SA, Kaur S, Rahman A, et al. Immunoproteomic identification and characterization of *Leishmania* membrane proteins as non-invasive diagnostic candidates for clinical visceral leishmaniasis. Sci Rep. 2018;8(1):12110.doi.org/10.1038/s41598-018-30546-y

Mahdavi R, Fadaei R, Baradaran B, et al. Development of a novel enzyme-linked immunosorbent assay and lateral flow test system for improved serodiagnosis of visceral leishmaniasis in different areas of endemicity. Microbiol Spectr. 2023;11(3).doi.org/10.1128/spectrum.04338-22

Maia RC. Seleção de potenciais peptídeos para o diagnóstico sorológico da leishmaniose visceral canina por imunoinformática [Tese de Doutorado]. 2021.

Lage DP, Mendonça LZ, Santos AL, et al. A new *Leishmania*-specific hypothetical protein and its non-described specific B cell conformational epitope applied in the serodiagnosis of canine visceral leishmaniasis. Parasitol Res. 2016;115:1649-1658.doi.org/10.1007/s00436-016-4904

Lima MP, Barbosa HS, Guimarães FR, et al. Evaluation of a hypothetical protein for serodiagnosis and as a potential marker for post-treatment serological evaluation of tegumentary leishmaniasis patients. Parasitol Res. 2017;116:1197-1206.doi.org/10.1007/s00436-017-5397-y

Teixeira HC, Moreira G, Silva L, et al. Refinement of the rKLi8.3-based serodiagnostic ELISA allows detection of canine visceral leishmaniasis in dogs with low antibody titers. 2024.

Assis LM, Silva JO, Oliveira JS, et al. B-cell epitopes of antigenic proteins in *Leishmania infantum*: An in silico analysis. Parasite Immunol. 2014;36(7):313-323.doi.org/10.1111/pim.12111

Marlais T, Williams S, Kafi M, et al. Isolation and characterization of *Leishmania donovani* protein antigens from urine of visceral leishmaniasis patients. PLoS One. 2020;15(9).
